# An optimized protocol for total RNA isolation from archived formalin-fixed paraffin-embedded tissues to identify the long non-coding RNA in oral squamous cell carcinomas

**DOI:** 10.1016/j.mex.2021.101602

**Published:** 2021-12-10

**Authors:** Kiran Kumar, Ajaykumar Oli, Kaveri Hallikeri, A S Shilpasree, Mallikarjun Goni

**Affiliations:** a Department of Oral and Maxillofacial Pathology, SDM College of Dental Sciences and Hospital, (A Constituent Unit of Shri Dharmasthala Manjunatheshwara University), Dharwad, Karnataka 580009, India; b Department of Biomedical Science, SDM Research Institute for Biomedical Sciences, (A Constituent Unit of Shri Dharmasthala Manjunatheshwara University), Dharwad, Karnataka 580009, India; c Department of Biochemistry, SDM College of Medical Sciences and Hospital, (A Constituent Unit of Shri Dharmasthala Manjunatheshwara University), Dharwad, Karnataka 580009, India

**Keywords:** LncRNAs, Oral cancer, RNA extraction protocol, Paraffin-embedded tissues, qRT-PCR

## Abstract

Approximately 93% of the human genome is translated into RNAs, of which only 2% code for proteins and the rest 98% are noncoding RNAs. Long non-coding RNAs (lncRNAs) are a class of non-coding RNAs of > 200 nucleotides length that are emerging as novel players in the field of cancer diagnostics or prognostics. Recently, lncRNAs are known to be associated with oral squamous cell carcinomas (OSCC). The demonstration of stable lncRNA has been a challenge in formalin-fixed paraffin-embedded tissues (FFPE). The survivability and expression level of lncRNA in FFPE tissues compared with fresh tissues is not well documented in the literature. Hence, we designed the current pilot study with the main aim to optimize modified TRI (Total RNA isolation) reagent RNA isolation protocol to identify the lncRNA expression in archived FFPE tissues of OSCC in comparison to the standard RNA isolation kit method. The findings of our study demonstrated that the RNA quantity and quality were comparatively better with the optimized TRI reagent modified protocol than the standard RNA isolation kit method. Furthermore, ct (cycle threshold) values after reverse-transcription and qRT-PCR (Quantitative Real time PCR) were comparable and almost equal in both the methods for normal mucosa (control) and OSCC samples.

## Highlights

• TRI reagent modified protocol for RNA isolation is cost-effective compared to the kit method.
• The Quality and quantity of isolated RNA is better in the TRI reagent modified protocol compared to the kit method.
• The TRI reagent modified procedure has fewer steps than the kit method without requiring any additional training or time.

## Graphical abstract

**Figure:**
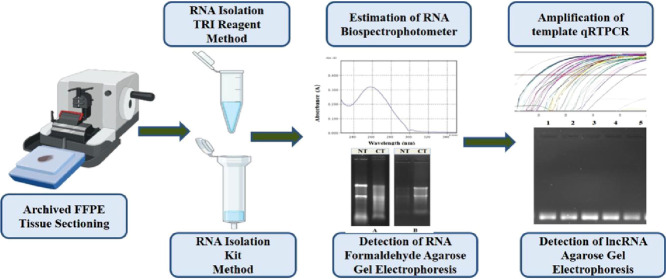

Specifications table

**Table:** 

Subject Area:	Biochemistry, Genetics and Molecular Biology
More specific subject area:	RNA isolation protocol from formalin fixed paraffin embedded tissues of oral squamous cell carcinomas to identify Long non-coding RNAs
Protocol name:	Modified TRI reagent RNA isolation protocol
Reagents/tools:	**Reagents and consumables:** 1. Surgical scalpel blade No. 22 (Lister, catalogue number: MM29101853), Stored at room temperature (RT). 2. Safe-Lock Eppendorf Tubes 1.5 ml (Eppendorf, catalogue number: 0030120.086), Stored at RT. 3. Safe-Lock Eppendorf Tubes 2.0 ml (Eppendorf, catalogue number: 0030120.094), Stored at RT. 4. epT.I.P.S. Eppendorf Tips 0.1-10 µl (Eppendorf, catalogue number: 0030073.002), Stored at RT. 5. epT.I.P.S. Eppendorf Tips 2-200 µl (Eppendorf, catalogue number: 0030073.061), Stored at RT. 6. epT.I.P.S. Eppendorf Tips 50-1,000 µl (Eppendorf, catalogue number: 0030073.100), Stored at RT. 7. Parafilm 2 Inch. X 250 foot Roll (Bemis, catalogue number: PM-992), Store at RT. 8. Formalin-Fixed Paraffin-Embedded (FFPE) tissues. 9. 2-Propanol (Sigma-Aldrich, catalogue number: 278475), Stored at RT. 10. Agarose (Sigma-Aldrich, catalogue number: A9539), Stored at RT. 11. Chloroform (HiMedia, catalogue number: MB109), Stored at RT. 12. Ethanol absolute (HiMedia, catalogue number: MB106), Stored at RT. 13. N,N,N′, N′-Tetramethylethylenediamine (Sigma-Aldrich, BioReagent, catalogue number: T9281), Stored at R11T. 14. Nuclease-Free Water (not DEPC-Treated) (ThermoFisher Scientific, Invitrogen, catalogue number: AM9932), Stored at 2–8 °C. 15. Proteinase K (HiMedia, catalogue number: MB086), Stored at 2–8 °C. 16. Sodium chloride (Sigma-Aldrich, BioXtra, catalogue number: S7653), Stored at RT. 17. Trizma hydrochloride (Merck Millipore, catalogue number: T5941), Stored at RT 18. Xylenes (Sigma-Aldrich, catalogue number: 247642), Stored at RT. 19. SYBR safe – Hi safe gel stain (HiMedia, catalogue number: ML053), Stored at 2–8 °C 20. Tris-Base (Sigma-Aldrich, catalogue number: T1503), Stored at RT. 21. MOPS (3-(N-morpholino) propane sulfonic acid) - (MP Biomedicals LLC, catalogue number-102370), Stored at RT. 22. Formaldehyde 37% (Sigma- Aldrich, Catalogue number: F8775-500ml), Stored at RT. 23. Bromophenol Blue dye (Sigma-Aldrich, Catalogue number: B0126-25G), Stored at RT. 24. Formamide puriss, P.A. ACS reagent ≥ 99.5% (Sigma-Aldrich, catalogue number: 47670-25 ml-F), Stored at RT. 25. RNase Kill (HiMedia, Catalogue number: ML162-250 ml), Stored at RT. 26. DEPC Diethyl Pyrocarbonate (HiMedia, Catalogue number: MB076–25 ml) stored at 4 °C. 27. Tri reagent (Sigma-Aldrich catalogue number: T9424-200 ml) stored at RT. 28. Ethidium bromide (HiMedia catalogue number: MB071-5G). 29. 0.2 M Phosphate Buffer Saline (PBS), pH 7.0 (see Recipes) Stored at 4 °C 30. Proteinase K Digestion Buffer (see Recipes), Stored at 4 °C 31. 2x RNA loading buffer (see Recipes), Stored at -20 °C. 32. 6x DNA loading buffer (see Recipes), Stored at 4 °C
	33. 10X MOPS buffer (see Recipes), Stored at 4 °C. 34. Formaldehyde Agarose Gel Mix (see Recipes). 35. 1x Gel MOPS running Buffer (see Recipes). 36. Oligonucleotide Primers (Eurofins Genomics), Stored at -20 °C. 37. Blue and Black Marker Pens (Kokuyo Camlin), Stored at RT. 38. NucleoSpin total RNA FFPE Kit (Lot.No.740982.10, Machery-Nagel-GmBH & Co.KG Germany). Stored at 4 °C.
	**Equipment:** 1. Pipettes: 6-pack (0.1–2.5 µl, 0.5–10 µl, 2–20 µl (Yellow), 10–100 µl, 20–200 µl, 100–1,000 µl) (Eppendorf, Model: Research Plus®). 2. Semi-automatic soft tissue microtome (Leica, Model: RM2245). 3. Thermomixer (Eppendorf, Model: Thermo Mixer C). 4. Dry block heater (Stuart, Model: SBH-200DC). 5. Centrifuge (Eppendorf, Model: 5424R). 6. Centrifuge (Eppendorf, Model: 5424). 7. Micro Centrifuge (Remi, Model: RM-02 Plus). 8. Vortex Mixer (Labnet, Model: S0200). 9. BioSpectrometer kinetic (Eppendorf, Model: 6136) 10. RockyMax Rocking shaker (Tarsons, Model:4080) 11. BioRad Molecular Imager XRS+ (BioRad, Hercules, CA, Serial number: 721BR14602). 12. Water bath (Grant, Model: SAPD). 13. Dry block Heater (Stuart, Model: SBH-200DC). 14. Biomedical Freezer - 20 ˚C (Panasonic, Model: MOF-U5312). 15. Blood Bank refrigerator (Panasonic, Model: MBR305GR). 16. Rotor Gene Q MDx (Qiagen, Model: 9002043). 17. Submarine Electrophoresis System (Takara model: Mupid one).
Experimental design:	*Pilot study*
**Trial registration**:	*Not Applicable*
Ethics:	*The Institutional Ethical Committee approval was obtained for the present study (Ref. No SDMIEC: 85:2020).*
Value of the Protocol:	• TRI reagent modified protocol for RNA isolation is cost-effective compared to the kit method. • The Quality and quantity of isolated RNA is better in the TRI reagent modified protocol compared to the kit method • The TRI reagent modified protocol has fewer steps than the kit method and does not require any additional training or time.

## Description of protocol

An optimized protocol for total RNA Isolation from Archived Formalin-fixed Paraffin-embedded Tissues to Identify the Long non-coding RNA (lncRNAs) in oral squamous Cell Carcinomas

## Introduction

The long non-coding RNAs (lncRNAs) are a class of RNAs >200 nucleotides length. These lncRNAs are emerging as novel players in the field of cancer diagnostics or prognostics as they are involved in oncogenic and tumor-suppressive regulatory functions [1]. Recently, lncRNAs dysregulation has been associated with oral squamous cell carcinomas (OSCC) and has been known to affect various aspects such as cellular homeostasis, proliferation, survival, migration, or genomic stability [2]. However, there is scarcity of literature reports on association of dysregulated lncRNAs with head and neck squamous cell carcinoma (HNSCC).The functional significance of lncRNAs specifically in OSCC has been remained unexplored [3].

The ability to predictably retrieve sufficient RNA for cDNA template generation and subsequent quantitative polymerase chain reaction (qPCR) facilitates differential gene transcriptional analysis [4]. Recent introduction of high-content, high-throughput Quantitative Real time PCR (qRT.-PCR) has demonstrated that RNA extracted from FFPE tissue sections could produce reliable qRT-PCR data [5,6]. Short RNA fragments like miRNA are stable and detectable in qRT-PCR in FFPE tissues. Literature reports have revealed the reliable expression levels of miRNA in FFPE as compared to paired fresh-frozen samples [7,8]. However, survivability and expression level of lncRNA in FFPE tissues as compared to fresh tissues is not well documented in the literature. This could be owing to the longer length, degradation, and fragmentation of lncRNA associated with fixation and processing [5].

With this background, we designed the current pilot study with the main aim to optimize the modified TRI reagent RNA isolation protocol to identify few important lncRNA expression in archived FFPE tissues of OSCC. Normal mucosa was used as control. In addition, we also aimed to compare commercially available column-based RNA isolation kit viz. NucleoSpin, Total RNA FFPE, Germany with modified TRI reagent RNA isolation protocol to check for the quality, and its usefulness in lncRNA expression analysis.

### RNA isolation procedure from FFPE by optimized TRI reagent modified protocol

#### 1. FFPE tissue sectioning

• Take FFPE tissues sections of 6-8 µ thickness using soft tissue microtome.
• Clean the blades with xylene after each sample to remove paraffin residues.
• Transfer 4 to 5 cut paraffin sections into 1.5 mL Eppendorf tubes.

**Note:** Slightly rolled up sections can be better handled and this could be obtained by decreasing the temperature of the paraffin blocks by placing ice on the cutting surface or putting paraffin blocks in a freezer before cutting.

#### 2. Deparaffinization

• Add 1 ml of xylene, vortex, and then incubate in the water bath at 56 ˚C for 10 min.
• Centrifuge the sample at 14,000 rpm for 10 min at room temperature and discard the supernatant
• Repeat the above procedure three times till the tissue sections are completely deparaffinized.
• Centrifuge again at 14,000 rpm for 2 min at room temperature and discard the supernatant completely. Avoid the residues of xylene.
• Add 1ml of absolute ethanol, vortex, and centrifuge at 14,000 rpm for 10 min at room temperature and discard the supernatant.
• Again, centrifuge the tubes at 14,000 rpm for 5 min at room temperature to remove the residues of ethanol.
• To wash the pellet with Phosphate Buffer-Saline (PBS), add 500 µl of PBS, vortex and then centrifuge the tubes at 14,000 rpm for 5 min at room temperature.
• Dry the pellet for 5 minutes at 37 ˚C in thermomixer or air dry till completely evaporation of traces of PBS.

**Note:** To remove the paraffin and unmask hidden or latent epitopes in preparation for downstream application. The procedure of Xylene and absolute alcohol can be repeated to ensure the complete removal of paraffin from tissue.

#### 3. Protein digestion

• Add proteinase K digestion buffer containing390 µl lysis buffer and 10 µl of proteinase K (500 µg/ml) and vortex it.
• Incubate the tube in the water bath at 56 ˚C for 60 min, after incubation immediately transfer the tube into the ice.
• Add 1ml TRIZOL reagent, vortex for 2–5 s, and incubate for 1-2 min at room temperature
• Add 0.2 ml of chloroform, vortex, and incubate at room temperature for 5 min
• Centrifuge at 13,000 rpm for 15 min at 4 °C and collect the supernatant.

**Note:** To digest proteins and remove contamination from nucleic acid preparations. Buffer was added in nucleic acid preparations for the inactivation of nucleases that could degrade RNA during isolation and purification applications. Carefully Removing the aqueous phase (supernatant) is a very crucial step and avoids contamination with the interphase and organic phase.

#### 4. RNA precipitation

• Add 0.6 ml of isopropanol and incubate at -20 °C overnight
• Following day, centrifuge at 13000 rpm at 4 °C for 15 mins
• Discard the supernatant and add 500 µl of 75% chilled ethanol
• Centrifuge at 10,000 rpm for 15 min at 4 °C
• Discard supernatant completely.

**Note:** Isopropanol precipitation is based on the principle of salting out, in the presence of salts that renders nucleic acid preferentially to become insoluble and the precipitate is collected by centrifugation. The process also purifies the RNA leaving out alcohol soluble salts, organic solvents, and detergents. The addition of glycogen was not done in the present protocol as it may cause contamination. Maintaining temperature during incubation and optimal centrifugal force is important to avoid degradation of RNA.

#### 5. Pellet drying

• Dry the pellet in thermomixer at 37 °C for 5min
• The RNA was eluted in 30–50 µL of nuclease-free water

**Note:** Ethanol should completely evaporate or else it prevents the RNA solubilization into nuclease-free water.

If the pellet dries out too much, the RNA crystallizes and is very difficult to resolubilize.

Main modifications made in the present optimized protocol compared to TRI reagent baseline protocol [**9**] are mentioned in Table 1.

**Table 1:** Comparison of old TRI reagent baseline protocol [9] and optimized TRI reagent modified protocol for RNA isolation:

Old TRI reagent protocol	Optimized TRI reagent modified protocol
**Deparaffinization**	
1 ml xylene to the sample and incubate at 50 °C for 3 min	Add 1ml of xylene to sample vertex and incubate at 56 ˚C for 10 min
**Protein digestion**	
protease K digestion buffer containing 500 μg/ml protease K to sample and incubate at 55 °C for 3 h.	protease K digestion buffer containing 500 μg/ml protease K to sample and incubate at 56 ˚C for 60 min
**RNA isolation & RNA precipitation**	
To aqueous phase, 10 μg glycogen is added and mixed. Total RNA is precipitated by mixing with 0.6 ml isopropyl alcohol at -20 °C for at least 1 h.	To the aqueous phase, Add 0.6 ml of isopropanol and incubate at -20 0 C for overnight for RNA precipitation.
**RNA wash, solubilization and Pellet Drying**	
RNA pellet is washed with 100% ethanol, briefly air-dried. Pallet is dissolved in RNase-free water	RNA pellet is washed in 75% chilled ethanol, dried in thermo mixer at 37 °C for 5 min. Pellet is dissolved in nuclease-free water

### RNA isolation procedure from FFPE by the kit method

RNA isolation was also carried out from the study samples using a column-based kit (NucleoSpin, Total RNA FFPE, Germany) following the manufacturer's protocol.

### Estimation of RNA concentration and quality

The concentration of RNA was estimated at 260:280 absorbance using a Bio-Spectrophotometer (Eppendorf, model no.6136, Germany) in nanograms. Then the RNA was stored in aliquots of the required quantity at -20 °C in Eppendorf tubes sealed with parafilm.

After the RNA quantification, the integrity of RNA was verified using 200 ng of RNA in 1% formaldehyde agarose gel electrophoresis in 1X MOPS buffer at 100 V for 30 min and stained with ethidium bromide (ETBR) to visualize the RNA bands. This helps to know the quality of the extracted RNA and also to determine the presence of any contaminants like DNA or Protein.

### cDNA synthesis

All RNA extracts were prepared at 1µg/µl per sample and transcribed into cDNA using Prime script 1^st^ strand cDNA synthesis kit (Takara, Japan) as per the manufacturer's instructions. DNA sample was diluted with nuclease-free water (1:10) and stored at -20 °C until further use.

### Quantitative real time PCR (qRT-PCR)

qRT-PCR (Rotor Gene Q MDx) was performed with TB green Mix (Takara, Japan) in a total volume of 20 µl. Primer sequence for lncRNAs and endogenous control gene (GAPDH) used to normalize the expression level were custom-designed (Table 2). Primers were designed using software Tool (https://bioinfo.ut.ee/primer3-0.4.0/) and were checked for specificity using basic local alignment search tool (https://blast.ncbi.nlm.nih.gov/Blast.cgi).The ct values of study cases were compared in TRI reagent modified protocol and kit method. Agarose gel electrophoresis was carried out to determine the molecular weight of the qRT-PCR end product**.**

**Table 2:** The primers sequence of lncRNAs and endogenous control gene.

Sl.No.	Name of the Primers	Sequences (5’- 3’)
**1.**	**HOTAIR-F**	GCAGTGGAATGGAACGGATT
	**HOTAIR-R**	ATCAGACTCTTTGGGGCCTT
**2.**	**MEG3-F**	TCCATGCTGAGCTGCTGCCAAG
	**MEG3-R**	AGTCGACAAAGACTGACACCC
**3.**	**H19-F**	AGACACCATCGGAACAGCAG
	**H19-R**	CTCTGGGATGATGTGGTGGC
**4.**	**MALAT1-F**	CCTACTGGGCTGACATTAACT
	**MALAT1-R**	GCCACTTCCTTTGCTCTGC
**5.**	**GAPDH-F**	GGGGAAGGTGAAGGTCGGAG
	**GAPDH-R**	ACGGTGCCATGGAATTTGCC

**Fig. 1:**
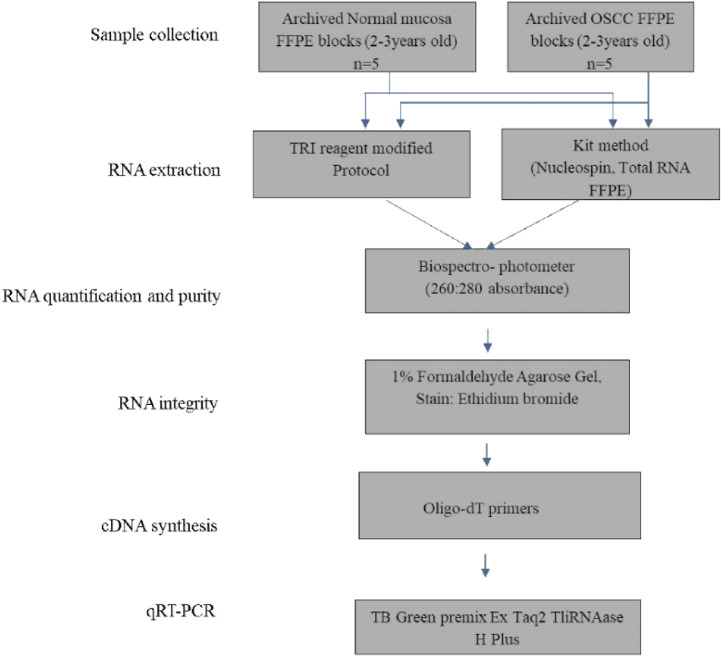
Flow chart of the steps carried out in the study.

The flow chart of the work carried out is represented in Fig. 1.

## Result validation

### Comparison of total RNA isolation methods (quantity & quality)

**Fig. 2:**
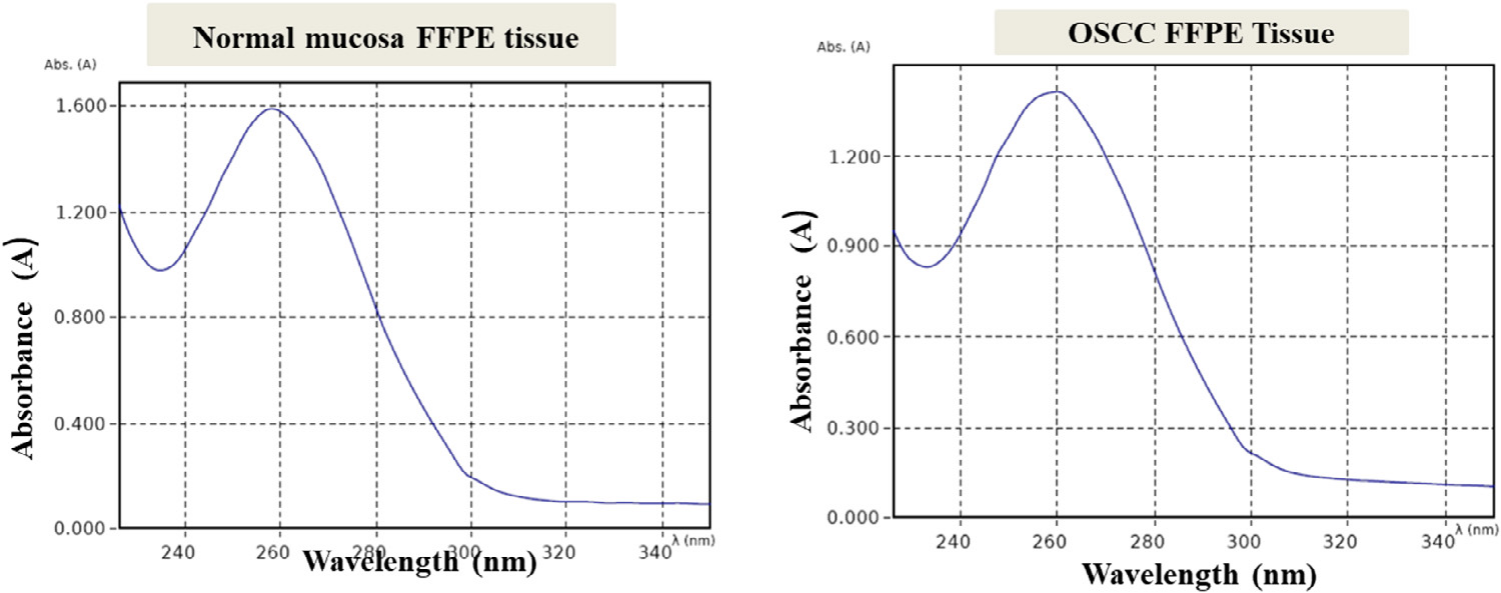
Graphs showing absorbance of RNA isolated from FFPE tissues of normal mucosa (Control) and OSCC at different wavelength with maximum absorbance at 260 nm.

**Fig. 3:**
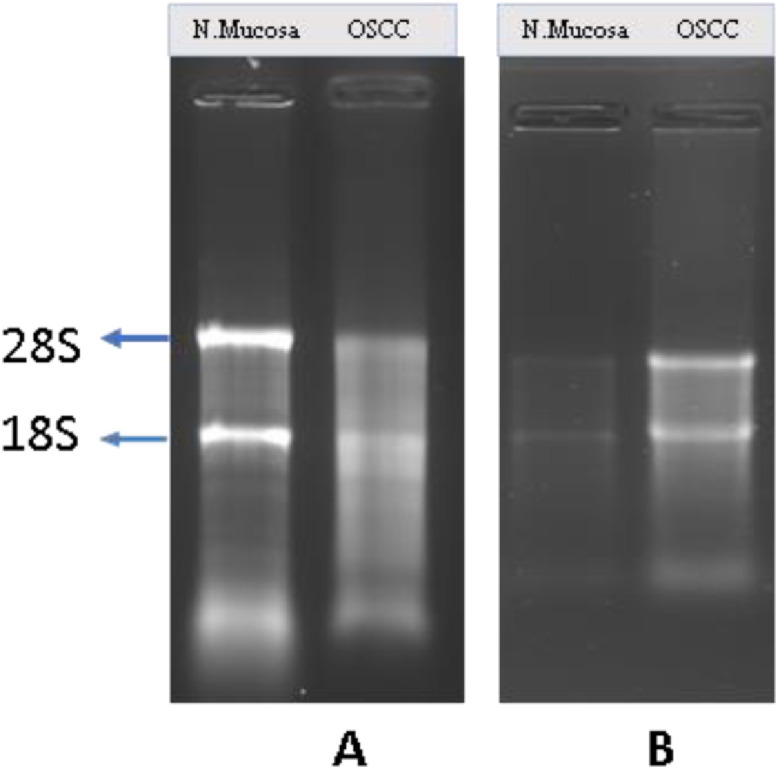
1% Formaldehyde Agarose Gel Electrophoresis of study samples by TRI reagent modified protocol and the kit method A (TRI reagent modified protocol)- Lane 1: Normal mucosa FPPE sample, Lane 2: OSCC FFPE sample B (Kit method) B- Lane 1: Normal mucosa FPPE sample, Lane 2: OSCC FFPE sample.

The total RNA concentrations using TRI reagent modified protocol was better than the kit method (Table 3). The A260:A280 ratio was between 1.71 to 1.92 indicated the good quality RNA in both RNA isolation methods (Table 3, Fig. 2). The integrity of RNA was verified by resolving in 1% formaldehyde agarose gel electrophoresis. Bands were visualized by staining in ethidium bromide stain (HiMedia, Mumbai). (Fig. 3).

**Table 3:** The baseline RNA concentrations of study cases measured byBioSpectrometer kinetic (Eppendorf, Model: 6136, Germany).

OSCC Samples - Kit Method					OSCC Samples – TRI-reagent Method				
Name of the sample	Concentration (ng/µl)	Absorbance	A260/280 ratio	A230/260 ratio	Name of the sample	Concentration (ng/µl)	Absorbance	A260/280 ratio	A230/260 ratio
6/20	224.568	0.256	1.83	0.34	6/20	346.405	0.856	1.90	0.78
130/19	280.153	0.704	1.89	0.34	130/19	215.027	0.708	1.84	0.34
408/18	271.767	0.254	1.81	0.54	408/18	291.989	0.458	1.80	0.53
278/18	419.096	1.012	1.71	0.87	278/18	666.325	1.618	1.87	0.95
107/19	266.017	0.364	1.75	0.89	107/19	298.914	0.207	1.78	0.73


Normal mucosa samples- Kit Method					Normal mucosa samples - TRI-reagent Method				
Name of the sample	Concentration (ng/µl)	Absorbance	A260/280 ratio	A230/260 ratio	Name of the sample	Concentration (ng/µl)	Absorbance	A260/280 ratio	A230/260 ratio
NP5	205.7	0.264	1.76	0.191	NP5	240.06	0.601	1.81	0.37
NP6	239.3	0.598	1.84	0.365	NP6	201.2	0.253	1.89	0.42
NP8	216.2	0.290	1.87	0.365	NP8	303.7	0.759	1.92	0.39
NP9	235.7	0.589	1.86	0.29	NP9	226.9	0.317	1.85	0.59
NP10	563.8	1.410	1.74	0.66	NP10	462.3	1.210	1.84	0.63

### qRT-PCR analysis and melting curve analysis

**Fig. 4:**
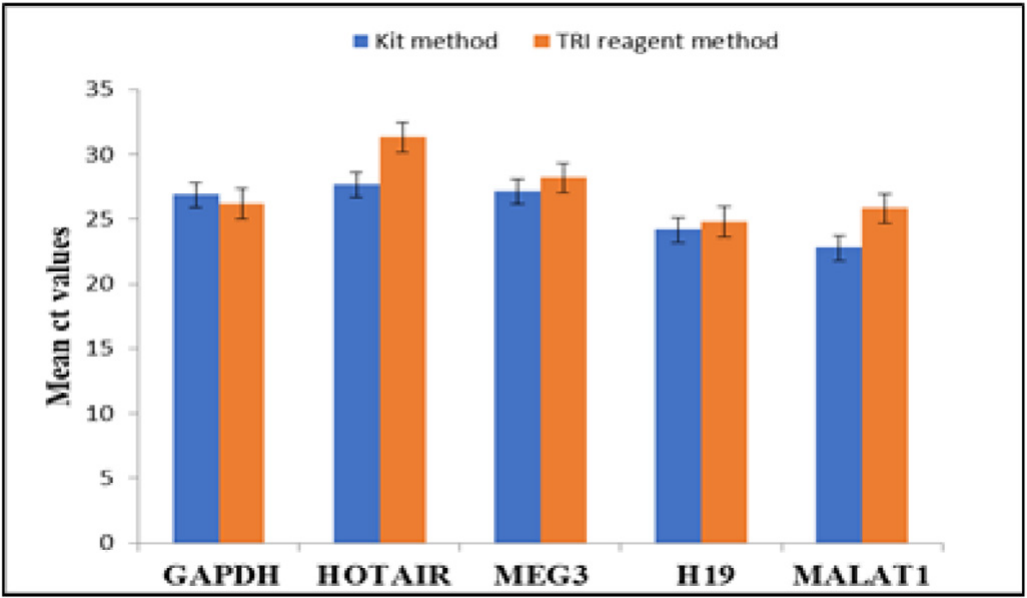
Graph showing comparative lncRNA mean ct values of OSCC samples where RNA isolation done in TRI reagent protocol and kit method.

**Fig. 5:**
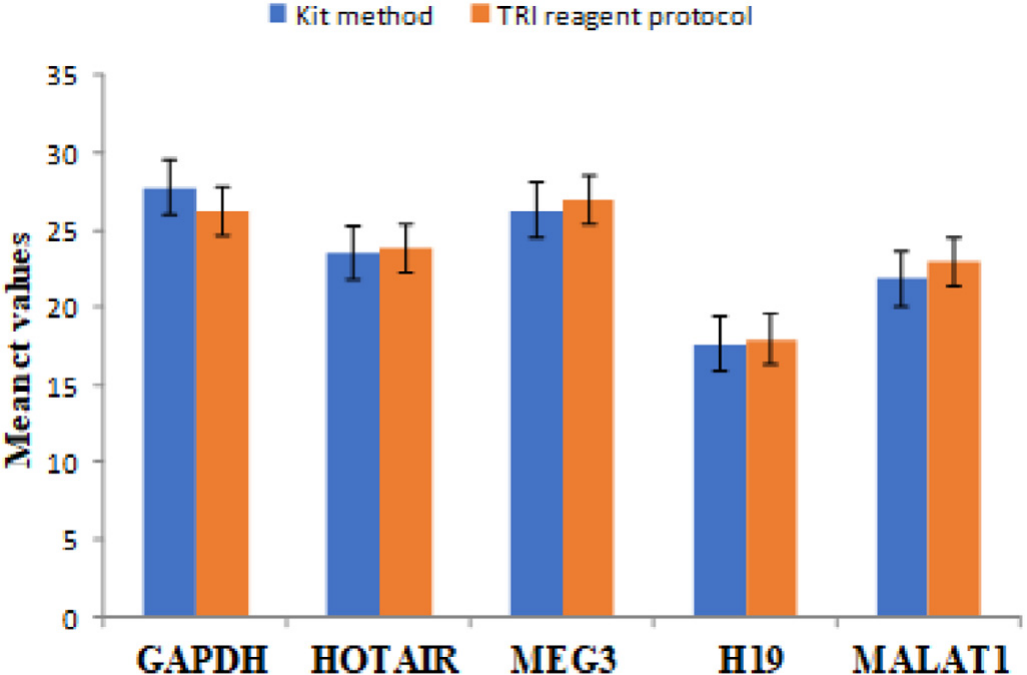
Graph showing comparative lncRNA mean ct values normal mucosa samples where RNA isolation done in TRI reagent protocol and kit method.

**Fig. 6:**
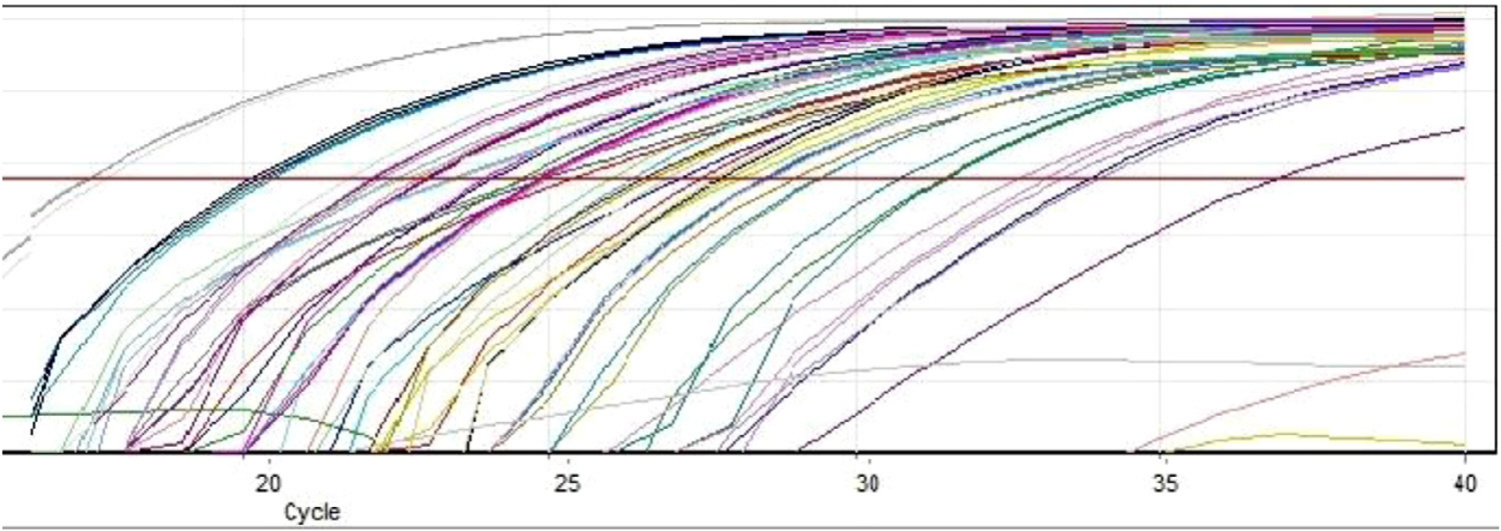
SYBR Green I assay for lncRNAs and Negative control reactions produced detectable amplicons after 40 PCR cycles.

**Fig. 7:**
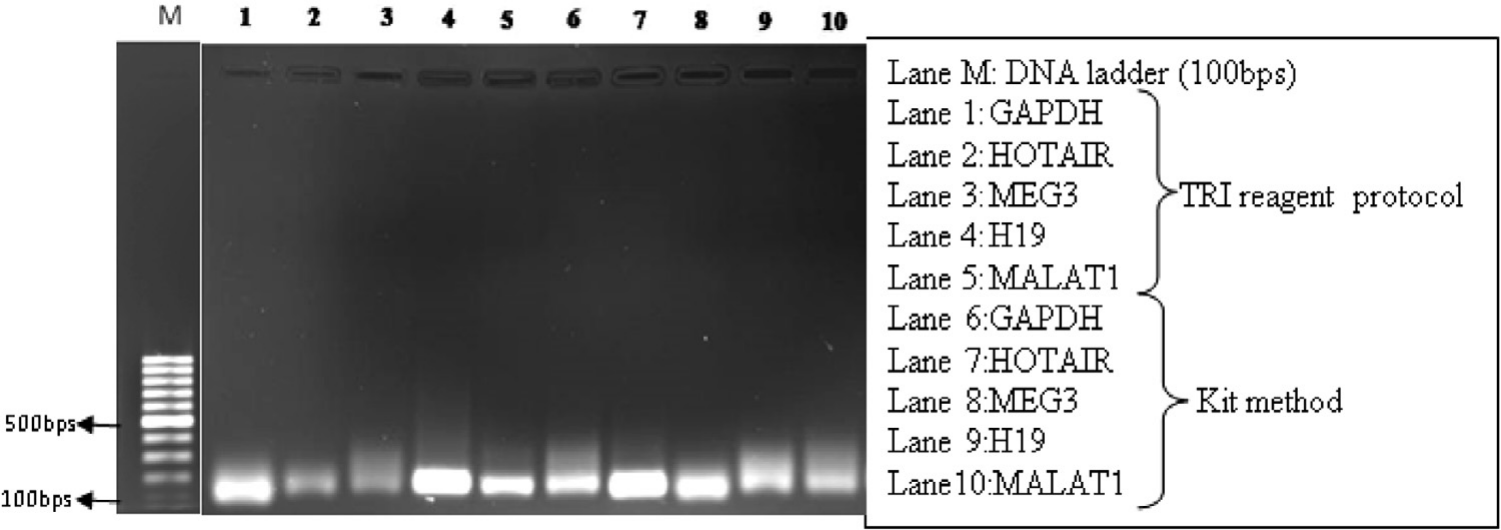
1% Agarose gel electrophoresis of qRT-PCR end product of normal mucosa FFPE samples indicating the expected size of amplicons between 100 to 200 bps.

**Fig. 8:**
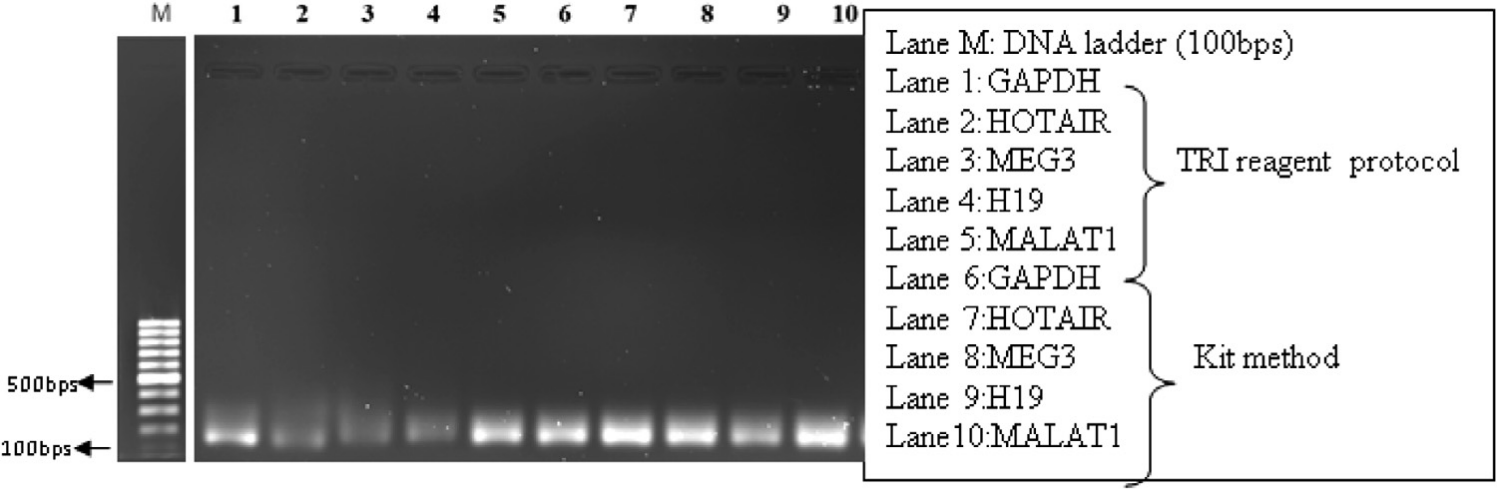
1% Agarose gel electrophoresis of qRT-PCR end product of OSCC FFPE samples indicating the expected size of amplicons between 100–200 bps.

The cyclic threshold values (ct values) of study cases in TRI reagent modified protocol and kit method were found to be comparable and the mean difference between them was < 1(Figs. 4 and 5). Melting curve analysis was carried out to assess the specificity of each primer pair (Fig. 6). Agarose gel electrophoresis of qRT-PCR product was carried out to confirm the molecular weight of lncRNAs which were < 200 nucleotide base pair units (Figs. 7 and 8).

### Conclusion

The present study results demonstrated that RNA quantity and quality was comparatively better with TRI reagent modified protocol than the kit method. The ct values after reverse-transcription and qRT-PCR were comparable and almost equal in both the methods for normal mucosa (control) and OSCC samples.

Furthermore, the TRI reagent modified RNA isolation approach was cost-effective, costing roughly 1/3 of the cost of the kit method, and is particularly advantageous when there are a large number of samples. Additionally, while the column-based kit method took less time to isolate RNA, it required more steps, which could lead to human error.

The TRI optimized protocol for RNA isolation effectively demonstrated lncRNA expression in oral tissues without demanding any additional training or extra time and also saves money. Hence, we recommend to use TRI optimized method for RNA isolation in lncRNA expression studies using FFPE tissues.

### Recipes

**0.2M Phosphate Buffer Saline (PBS), pH 7.0**

(a) Dissolve 27.8 g of monobasic sodium phosphate in 1 L deionized water – solution A
(b) Dissolve 53.65 g of dibasic sodium phosphate in 1 L deionized water – solution B

Mix 19.5 ml of solution A, 30.5 ml of solution B, 1 g of sodium chloride and adjust the volume to 100 ml with deionized water.

**Proteinase K Digestion Buffer (FFPE Tissue Dissolver)**

 Tris HCl – 20 mM (pH 8.0)
 CaCl_2_ – 1 mM
 SDS – 0.5 %
 Proteinase K – 500 µg/ml.

**2X RNA Loading Dye**

 Formamide Ultrapure- 95%
 EDTA (pH 8.0) – 5 mM
 Bromophenol Blue – 0.025%
 Ethidium Bromide – 10 µg/ml.

**6X DNA Loading Dye**

 Tris Base – 10 mM (pH 8.0)
 EDTA – 50 mM (pH 8.0)
 Glycerol – 30%
 Bromophenol Blue – 0.025%

**10X MOPS Buffer**

 MOPS – 200 mM (pH 7.0)
 EDTA – 10 mM
 Sodium Acetate – 50 mM
 Note: Adjusted the pH with KOH

**Formaldehyde Agarose Gel Mix**

 Agarose – 1 gm
 10x MOPS buffer - 10 ml
 Distilled Water – 72 ml

Note: Melt agarose in the oven, then let it to cool at 55 °C, then add 18 ml of Formaldehyde

(37%).

**1X Gel MOPS running Buffer**

 10 X MOPS buffer – 100 ml
 37% Formaldehyde – 20 ml
 Distilled water – 880 ml
 Total volume – 1000 ml
 Note: Make up a fresh buffer for each gel

## Declaration of Competing Interest

The authors declare that they have no known competing financial interests or personal relationships that could have appeared to influence the work reported in this paper.
